# Free Convection in a Square Ternary Hybrid Nanoliquid Chamber with Linearly Heating Adjacent Walls

**DOI:** 10.3390/nano13212860

**Published:** 2023-10-28

**Authors:** Vemula Rajesh, Mikhail Sheremet

**Affiliations:** 1 Department of Mathematics, GITAM (Deemed to Be University), Hyderabad Campus, Hyderabad 502329, Telangana, India; v.rajesh.30@gmail.com; 2 Laboratory on Convective Heat and Mass Transfer, Tomsk State University, 634050 Tomsk, Russia

**Keywords:** square cavity, finite element method, numerical simulations, ternary hybrid nanofluid, heat transfer

## Abstract

In this study, mathematical modeling of the energy transfer and flow characteristics of ternary nanoliquid in a square enclosure is performed. In the cavity considered, the left and bottom borders are warmed uniformly or non-uniformly when the rest of the borders are cooled. The robust finite element method with quads and triangles as elements is used to work out the control equations of the problem. The current study is validated against previously published works, and good agreement is shown. The isolines are investigated for various Rayleigh numbers at uniform and non-uniform thermal boundary conditions. The impact of ternary hybrid nanofluids on the mean Nusselt number at hot borders is explored in dependence on the Rayleigh number and nanoparticle concentration. A comparative study of different fluids for the mean Nusselt number at heated borders is also conducted and analyzed with appropriate graphs and tables. It has been shown that ternary nanofluids can be more effective compared to mono- and hybrid nanofluids, with a more essential growth of the energy transport rate with nanoadditives concentration.

## 1. Introduction

The phenomenon of buoyancy-induced energy transport in enclosures has been the subject of a huge amount of study in the past, and it is an essential component for a diverse range of technological purposes. This is the case, for example, with the cooling of electronic systems, where a huge number of high-power dissipation units are frequently packaged in cabinets to minimize the amount of space required as well as the number of external cooling sources. In addition, heat transfer engineers often choose not to make use of mechanical devices for the circulation of the coolant because of the high amount of power it consumes, the excessive working noise it creates, or worries regarding the system’s reliability. On the other hand, the efficiency of thermal energy removal from equipment whose temperature management depends on thermal convection is typically severely limited by the poor thermal conductivity of standard thermal fluids like water, ethylene glycol, and mineral oils. Nanofluids, or liquid suspensions containing tiny solid particles, offer a potential solution to this issue because of their effective thermal conductivity, which is considerably greater when compared to the pure host fluid.

In their crucial work, Mahmoudi et al. [1] performed a meticulous simulation of the intricate phenomenon of free convection cooling. Specifically, they focused on the captivating scenario of two heat sources that were affixed in a vertical orientation to the horizontal walls of a confined enclosure. The study’s findings revealed a significant correlation between the Rayleigh numbers and the heater’s location with respect to the flow field and thermal patterns in the chamber. The mathematical modeling performed by Cho et al. [2] explored the thermal convection parameters of Al_2_O_3_–H_2_O nanoliquid within an enclosure. The cavity was defined by vertical isothermal borders featuring a complicated wavy sheet, while the horizontal borders remained straight and subjected to adiabatic conditions. The findings elucidated in this investigation offer valuable perspectives on potential methodologies to augment the convective thermal energy transport efficiency inside the enclosure featuring intricate, undulating border surfaces. Abu-Nada and Chamkha [3] conducted a computational investigation of the steady mixed convection phenomenon in a lid-driven chamber. The working fluid considered was a nanosuspension with CuO nanoadditives dispersed in water. The bottom surface of the chamber was characterized by a wavy pattern. The enclosure was thermally isolated along its left and right boundaries, while the undulating horizontal boundaries were held at fixed temperatures. The uppermost border was identified as the thermally active boundary, functioning as the heated enclosure boundary while simultaneously exhibiting a uniform and unchanging velocity. As a result, it has been observed that the inclusion of nanoparticles induces a notable enhancement in thermal energy transport for various Richardson numbers and ratios of bottom wall geometry. The numerical solution presented by Sheremet et al. [4] pertains to the MHD thermal convection of a Cu–H_2_O nanoliquid within an irregular, porous, semi-open chamber. The chamber contains a heat source that maintains a constant temperature. The solution was obtained by employing the finite difference technique. The researchers have observed an enhancement in energy transfer with a rise in the Rayleigh number and a decrease in thermal energy transport with a rise in the Hartmann number. The numerical investigation conducted by Mondal and Mahapatra [5] focuses on magnetohydrodynamics (MHD) mixed convection flow and mass transport of Al_2_O_3_–H_2_O nanoliquid within a trapezoidal chamber. The study specifically examines the presence of discrete energy and mass sources placed at the lower border of the chamber. The present investigation has observed that the combined convective flow under a weak magnetic effect and within a chamber of low aspect ratio and rectangular shape consistently yields favorable results in terms of minimizing total entropy production. Furthermore, it has been determined that the central region of the bottom border of the chamber is particularly well-suited for the introduction of thermal energy and mass, ensuring a uniform distribution throughout the entirety of the cavity. In their numerical investigation, Reddy and Sreedevi [6] examined the impact of radiation on the transport of thermal energy and mass in a nanoliquid within a square cavity. This analysis was conducted by incorporating Buongiorno’s mathematical model. Thermophoresis and Brownian diffusion are employed for analysis. In this investigation, it has been observed that the strength of the thermal energy transfer rises with an increase in the Rayleigh number. A few other significant contributions using nanofluids to enhance heat transfer can be found in Xue et al. [7], Wang et al. [8], Qi et al. [9], Wang et al. [10], and Ge et al. [11]. Additionally, important investigations related to the cavity flow can be found in Chen et al. [12] and Luo and Yang [13].

Hybrid nanoliquids represent a novel class of working liquids that have been meticulously designed to possess augmented physical characteristics. The hybrid nanoliquids leverage the physical characteristics inherent in multiple varieties of nanoparticles. Recently, researchers have employed a hybrid nanofluid, consisting of a combination of disparate nanoparticles dispersed within a host fluid, as the operational medium to further augment the heat transfer properties. Henceforth, Mehryan et al. [14] undertook an investigation on the phenomenon of free convective thermal energy transport pertaining to the hybrid nanoliquid composed of Al_2_O_3_–Cu–H_2_O within a chamber that has been occupied by a porous medium. The experimental findings demonstrated a decrease in thermal conductivity through the implementation of nanoparticles within a permeable medium. The observed diminution of the thermal energy transport strength exhibits a significantly greater magnitude for the hybrid nanoliquid compared to the mono-nanoliquid. Alsabery et al. [15] conducted an investigation on the conjugate free convection and entropy production of Cu–Al_2_O_3_/H_2_O hybrid nanoliquids. The study was carried out inside a partially divided chamber featuring wavy and cold sidewalls. The experimental results have demonstrated that the utilization of hybrid nanofluids results in a growth in the Nusselt number in comparison with mono-nanofluids. Tayebi and Chamkha [16] employed a hybrid nanofluid to investigate the entropy production resulting from MHD thermal convection within a square chamber. The study considered the inclusion of an irregular circular conductive cylinder, accounting for the presence of irreversibilities arising from the magnetic effect. The model proposed by Khan et al. [17] examines heat management and thermal enhancement within a split lid-driven square chamber. In this investigation, a Y-shaped obstruction is positioned within the enclosure, which is saturated with hybrid nanoliquids composed of Al_2_O_3_, Cu, and water. The observation is made that a Y-shaped obstruction results in an augmentation in the strength of thermal energy transport within the enclosed space. Belhaj and Ben-Beya [18] conducted a study with the objective of analyzing the entropy production and MHD thermal convection of a hybrid nanoliquid in a chamber. The enclosure contained a warmed elliptical body positioned at its center, and a periodic-variable magnetic influence was present during the investigation. Several notable investigations on mono- and hybrid nanoliquids have been performed by Rajesh et al. [19], Sarkar et al. [20], Rajesh et al. [21], Sidik et al. [22], Dhinesh Kumar and Valan Arasu [23], Vemula et al. [24], and Sheremet et al. [25].

The investigation of thermal energy transfer in tri-hybrid nanofluids, wherein three distinct nanoparticles are dispersed within a base fluid, has garnered significant attention in recent studies. The process of synthesizing nanofluids is undertaken with the objective of augmenting the physical attributes inherent to said nanofluids. This methodology facilitates the generation of a heat carrier exhibiting augmented thermal and rheological properties. The experimental outcomes have shown that the heat conductivity of tri-hybrid nanofluid surpasses that of both mono-phased and double-phased nanofluids. In the investigation conducted by Sahoo [26], a novel coolant for radiators was examined. This coolant consisted of a ternary hybrid nanofluid containing nanoparticles of different shapes, specifically spherical (Al_2_O_3_), cylindrical (CNT), and platelet (graphene). The influence on heat transfer performance combined with the exergetic study, resulting from variations in coolant flow strength, the volume fraction of nanoadditives, and air velocity, has led to the conclusion that alterations in nanoadditives concentrations have an essential influence on heat transport performance, primarily owing to the morphology of the nanoadditives. Zahan et al. [27] conducted research on a ternary nanosuspension contained in distilled water and ethylene glycol, incorporating Co, Ag, and Zn nanoadditives at varying solid volume fractions. The objective was to examine the liquid dynamics and heat behavior within a CD nozzle. In this investigation, the enhanced strength of heat transport is determined by employing laminar-shaped nanoadditives. The investigation conducted by Shao et al. [28] involved the study of thermal convection in a prismatic chamber containing two mobile hot obstacles and porous material. The enclosure was saturated with a ternary hybrid nanoliquid. The findings suggest that a rise in the Darcy number characterizes a significant enhancement of both vertical and horizontal velocities, with improvements of 88.51% and 83.77%, respectively. Additionally, there is a substantial increase of 88.12% in the streamline patterns, as well as notable enhancements in Nusselt numbers and isotherms. The article by Sneha et al. [29] elucidates the dynamics of an unsteady flow involving a couple of stresses through the implementation of a ternary hybrid nanosuspension on a stretching sheet accompanied by porous material. Few other significant works using ternary hybrid nanofluids are presented in papers by Hafeez et al. [30], Adnan and Ashraf [31], Al Oweidi et al. [32], Puneeth et al. [33], and Mahmood and Khan [34].

Based on the comprehensive analysis of the existing literature, it becomes apparent that numerous investigations have been conducted thus far pertaining to the phenomenon of natural convection occurring within an enclosed space, employing both conventional fluids, nanofluids, and hybrid nanofluids. However, research on the thermal convection phenomena occurring within a confined space utilizing a ternary hybrid nanofluid has been relatively limited. In light of heat transfer applications in the realm of engineering and technology, the current investigation seeks to unveil the fundamental influence of a ternary hybrid nanofluid on the circulation and thermal energy transport properties inside a square chamber. Specifically, the left and bottom borders of the cavity are subjected to uniform or linear heating, while the rest are subjected to cooling. The analysis of the Nusselt number at the left and lower walls, combined with the variations in velocity and temperature within the cavity for different fluids, is conducted using FEM in COMSOL Multiphysics software 6.1. This investigation includes the influence of important governing factors such as the Rayleigh number and the nanoadditives concentration. The results are shown through the utilization of streamlines, isotherms, and other appropriate graphical representations and tabulated data.

## 2. Mathematical Analysis

**Figure 1:**
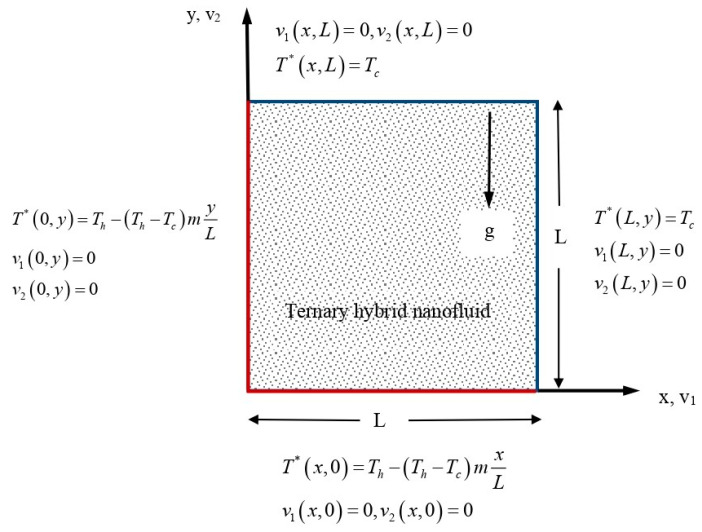
Problem model and coordinate system.

In our analysis, we shall consider a square chamber with a characteristic size of *L*, as depicted in Figure 1. When the right and top borders are subjected to cooling, the left and bottom walls experience either uniform or non-uniform heating.

The working liquid employed in this research is the ternary hybrid nanofluid, specifically composed of Cu–CuO–Al_2_O_3_ nanoparticles dispersed in water. It is an incompressible Newtonian medium with constant characteristics. Moreover, the present investigation neglects the influence of radiation and viscous dissipation while assuming the absence of internal heat generation/absorption. The problem has been analyzed using the two-dimensional Navier-Stokes equations and energy equation, considering steady and laminar flow conditions. The density variations in the nanosuspension are studied via the Boussinesq approach. It is postulated that the fundamental liquid, namely, water, and the nanoadditives are in a state of heat equilibrium. The control equations, given the aforementioned assumptions, can be expressed as

(1) $$\frac{\partial v_{1}}{\partial x}+\frac{\partial v_{2}}{\partial y}=0$$

(2) $$\rho_{thnf}\left(v_{1}\frac{\partial v_{1}}{\partial x}+v_{2}\frac{\partial v_{1}}{\partial y}\right)=-\frac{\partial p}{\partial x}+\mu_{thnf}\left(\frac{\partial^{2}v_{1}}{\partial x^{2}}+\frac{\partial^{2}v_{1}}{\partial y^{2}}\right)$$

(3) $$\rho_{thnf}\left(v_{1}\frac{\partial v_{2}}{\partial x}+v_{2}\frac{\partial v_{2}}{\partial y}\right)=-\frac{\partial p}{\partial y}+\mu_{thnf}\left(\frac{\partial^{2}v_{2}}{\partial x^{2}}+\frac{\partial^{2}v_{2}}{\partial y^{2}}\right)+{\left(\rho \beta \right)}_{thnf}g\left(T^{*}-T_{c}\right)$$

(4) $$v_{1}\frac{\partial T^{*}}{\partial x}+v_{2}\frac{\partial T^{*}}{\partial y}=\frac{\kappa_{thnf}}{{\left(\rho c_{p}\right)}_{thnf}}\left(\frac{\partial^{2}T^{*}}{\partial x^{2}}+\frac{\partial^{2}T^{*}}{\partial y^{2}}\right)$$

with boundary conditions

(5) $$\begin{array}{l} v_{1}\left(0,y\right)=0,v_{1}\left(L,y\right)=0,v_{1}\left(x,0\right)=0,v_{1}\left(x,L\right)=0,\\ v_{2}\left(0,y\right)=0,v_{2}\left(L,y\right)=0,v_{2}\left(x,0\right)=0,v_{2}\left(x,L\right)=0,\\ T^{*}\left(x,0\right)=T_{h}-\left(T_{h}-T_{c}\right)m\frac{x}{L},T^{*}\left(x,L\right)=T_{c},T^{*}\left(0,y\right)=T_{h}-\left(T_{h}-T_{c}\right)m\frac{y}{L},T^{*}\left(L,y\right)=T_{c} \end{array}$$

The physical features of host liquid, nanoadditives, and ternary hybrid nanosuspension are provided in Table 1 and Table 2.

Employing the mentioned transformations

(6) $$X=\frac{x}{L},Y=\frac{y}{L},V_{1}=\frac{v_{1}L}{\alpha_{f}},V_{2}=\frac{v_{2}L}{\alpha_{f}},P=\frac{pL^{2}}{\rho_{f}\alpha_{f}{}^{2}},T=\frac{T^{*}-T_{c}}{T_{h}-T_{c}}$$

into Equations (1)–(4) above, we obtain

(7) $$\frac{\partial V_{1}}{\partial X}+\frac{\partial V_{2}}{\partial Y}=0$$

(8) $$\frac{\rho_{thnf}}{\rho_{f}}\left(V_{1}\frac{\partial V_{1}}{\partial X}+V_{2}\frac{\partial V_{1}}{\partial Y}\right)=-\frac{\partial P}{\partial X}+Pr\frac{\mu_{thnf}}{\mu_{f}}\left(\frac{\partial^{2}V_{1}}{\partial X^{2}}+\frac{\partial^{2}V_{1}}{\partial Y^{2}}\right)$$

(9) $$\frac{\rho_{thnf}}{\rho_{f}}\left(V_{1}\frac{\partial V_{2}}{\partial X}+V_{2}\frac{\partial V_{2}}{\partial Y}\right)=-\frac{\partial P}{\partial Y}+Pr\frac{\mu_{thnf}}{\mu_{f}}\left(\frac{\partial^{2}V_{2}}{\partial X^{2}}+\frac{\partial^{2}V_{2}}{\partial Y^{2}}\right)+Ra\;Pr\frac{{\left(\rho \beta \right)}_{thnf}}{{\left(\rho \beta \right)}_{f}}T$$

(10) $$\frac{{\left(\rho c_{p}\right)}_{thnf}}{{\left(\rho c_{p}\right)}_{f}}\left(V_{1}\frac{\partial T}{\partial X}+V_{2}\frac{\partial T}{\partial Y}\right)=\frac{\kappa_{thnf}}{\kappa_{f}}\left(\frac{\partial^{2}T}{\partial X^{2}}+\frac{\partial^{2}T}{\partial Y^{2}}\right)$$

with conditions

(11) $$\begin{array}{l} V_{1}\left(0,Y\right)=0,V_{1}\left(1,Y\right)=0,V_{1}\left(X,0\right)=0,V_{1}\left(X,1\right)=0,\\ V_{2}\left(0,Y\right)=0,V_{2}\left(1,Y\right)=0,V_{2}\left(X,0\right)=0,V_{2}\left(X,1\right)=0,\\ T\left(X,0\right)=1-mX,T\left(X,1\right)=0,T\left(0,Y\right)=1-mY,T\left(1,Y\right)=0 \end{array}$$

Here $Ra=\frac{g\beta_{f}\left(T_{h}-T_{c}\right)L^{3}}{\upsilon_{f}\alpha_{f}}$ is the Rayleigh number and $Pr=\frac{\upsilon_{f}}{\alpha_{f}}$ is the Prandtl number.

The paramount parameter of technical significance in this investigation is the mean Nusselt number, as expressed by

$Nu_{L}=-\frac{\kappa_{thnf}}{\kappa_{f}}\left(\frac{2}{2-m}\right)\int_{0}^{1}{\left(\frac{\partial T}{\partial X}\right)}_{X=0}dY$ is the Nusselt number at the left border and

$Nu_{B}=-\frac{\kappa_{thnf}}{\kappa_{f}}\left(\frac{2}{2-m}\right)\int_{0}^{1}{\left(\frac{\partial T}{\partial Y}\right)}_{Y=0}dX$ is the Nusselt number at the bottom border.

## 3. Numerical Solution

**Figure 2:**
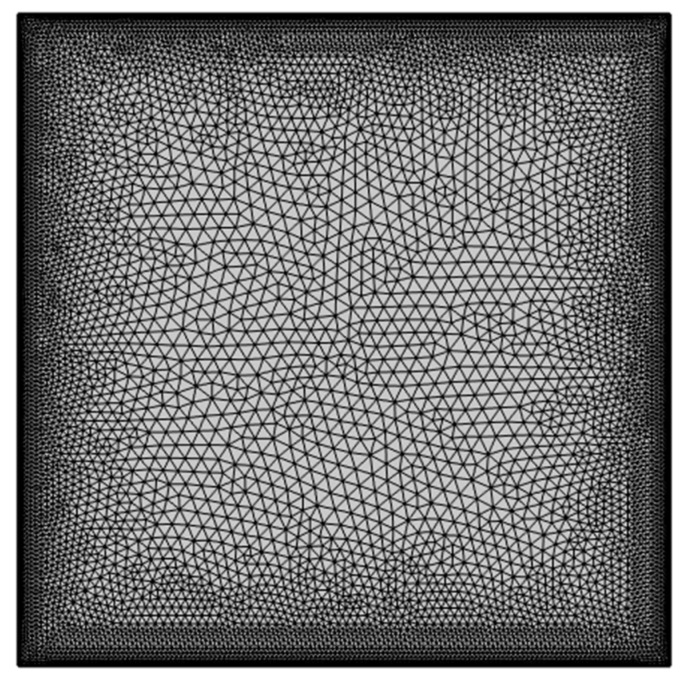
Computational mesh was selected for the study.

**Figure 3:**
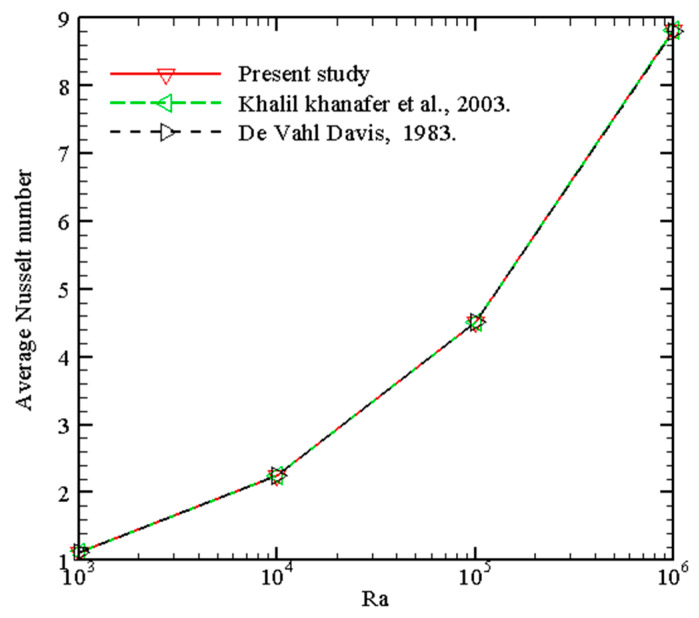
Validation with average Nusselt number [36,37].

**Figure 4:**
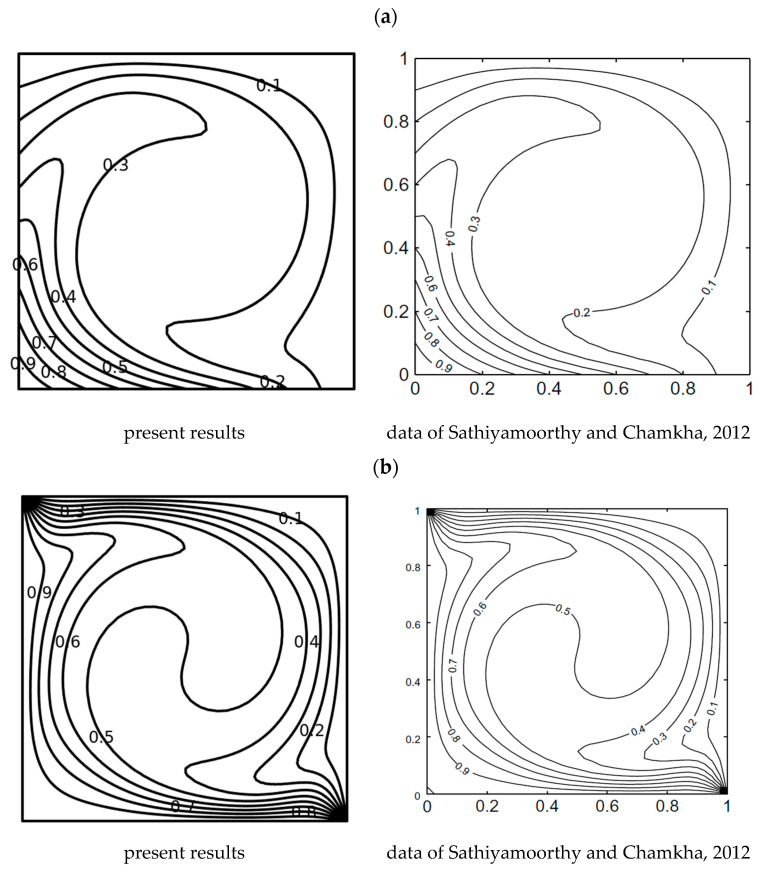
Comparison of isotherms for *Pr* = 0.054 and *Ra* = 10^5^. (**a**) Isotherms for *m* = 1; (**b**) Isotherms for *m* = 0 [38].

The PDEs (7)–(10) with conditions (11) modeling the problem have been unraveled employing the finite element method with COMSOL Multiphysics. The extra-fine mesh containing 16,946 elements is selected as the optimum mesh based on a grid-independent test, the details of which are provided in Table 3a,b. The mesh statistics of the selected effective mesh type (extra fine) are provided in Table 4. The computational mesh chosen for the present research is shown in Figure 2. Further, to validate the current model, the mean Nusselt numbers of this study for different *Ra* when *Pr* = 0.71, *b*1 = 0, *b*2 = 0, and *b*3 = 0 were compared to the findings in [36,37] with the same boundary conditions in Figure 3 and found excellent agreement between the results. Figure 4 shows a good agreement with data of Sathiyamoorthy and Chamkha [38]. This further confirms the accuracy of the current numerical results.

## 4. Results and Discussion

In the current investigation, we maintained *Ra* between 10^3^ and 10^5^, *Pr* at a value of 6.2, and the volume fractions of Al_2_O_3_, CuO, and Cu nanoadditives (*b*1, *b*2, and *b*3, respectively) at 0.04 each. Additionally, we considered two cases: *m* = 0 for constant/uniform heating and *m* = 1 for linear/non-uniform heating. These values were held constant throughout the study, unless explicitly stated otherwise for the specific parameter under examination.

**Figure 5:**
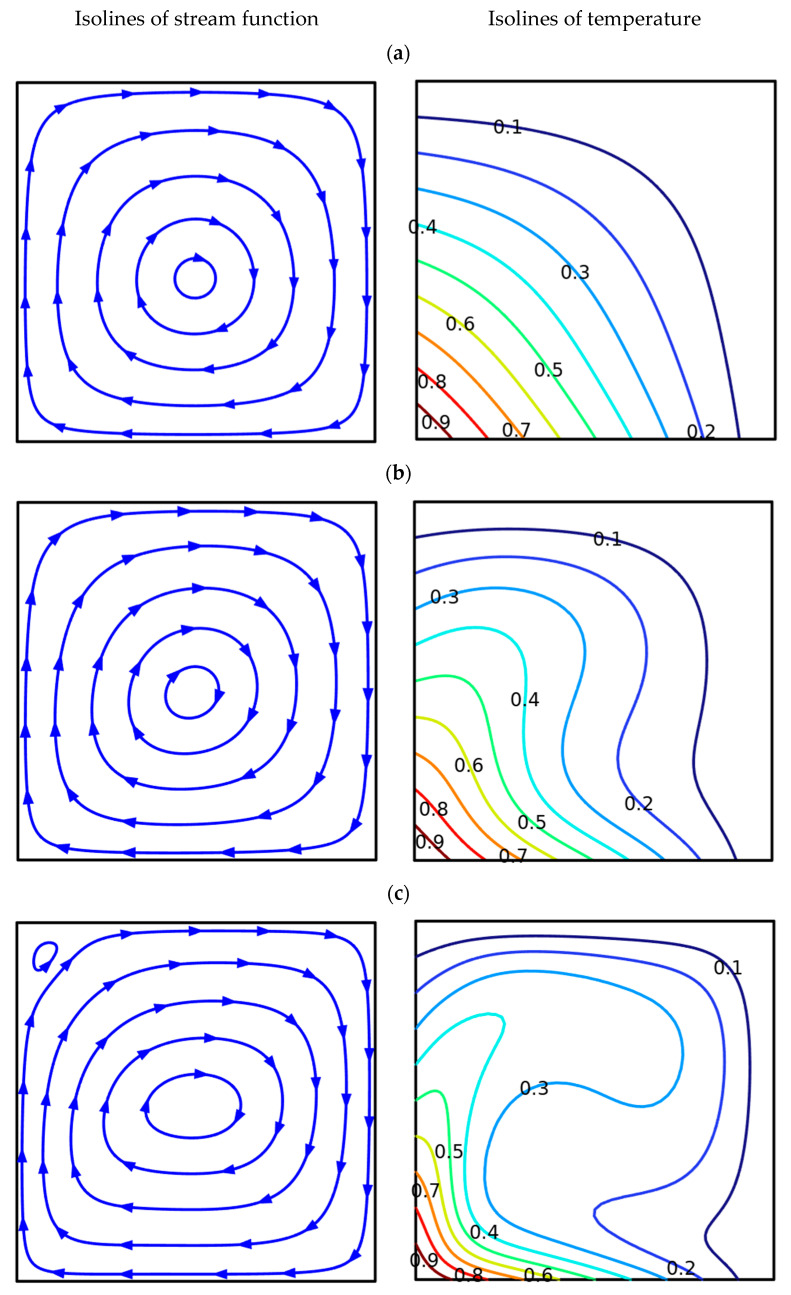
Isolines at *b*1 = *b*2 = *b*3 = 0.04, when *m* = 1 (linear heating). (**a**) *Ra* = 10^3^; (**b**) *Ra* = 10^4^; (**c**) *Ra* = 10^5^.

**Figure 6:**
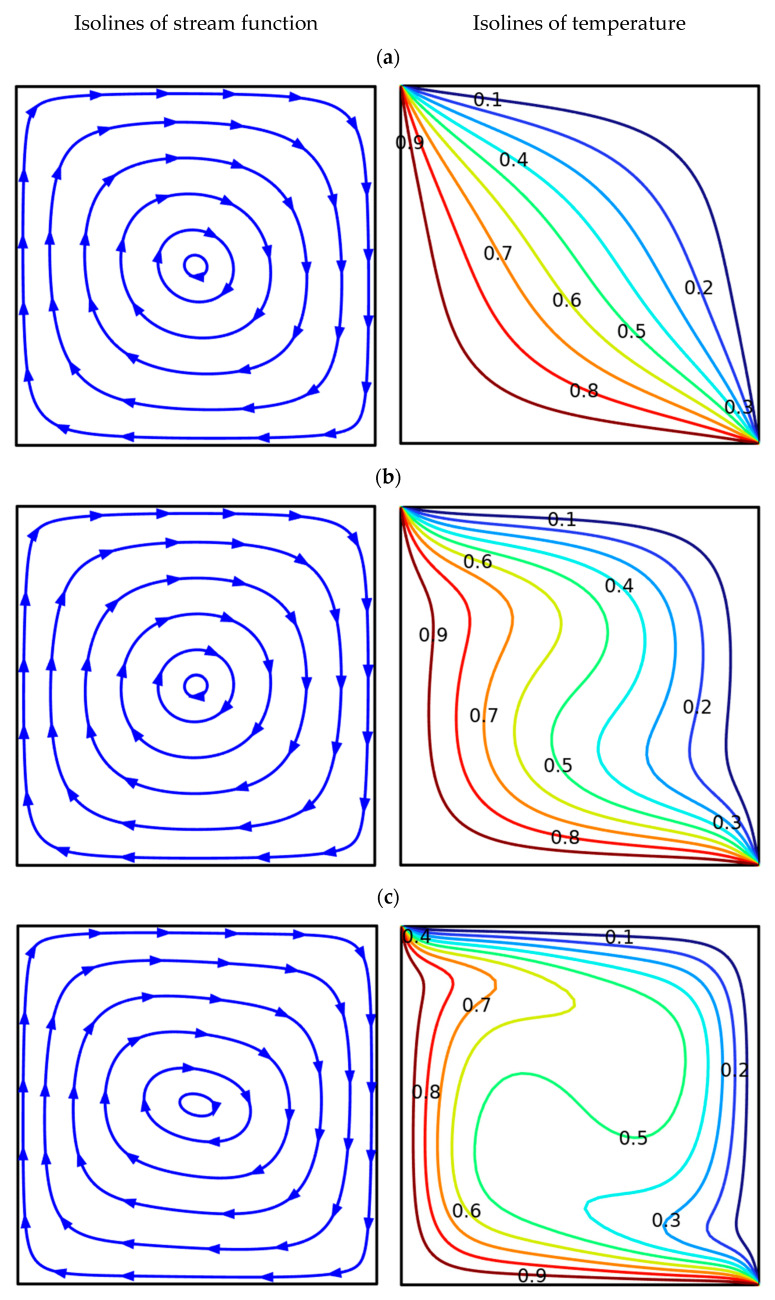
Isolines at *b*1 = *b*2 = *b*3 = 0.04, *m* = 0 (uniform heating). (**a**) *Ra* = 10^3^; (**b**) *Ra* = 10^4^; (**c**) *Ra* = 10^5^.

Figure 5 and Figure 6 present isolines for various Rayleigh numbers at uniform and non-uniform heating of the left and lower borders. For the non-uniform heating (see Figure 5), when the temperature on the left and lower borders is changed owing to the linear function of the coordinate, one can see an appearance of global clockwise flow and heating of the chamber from these walls. For a low Rayleigh number (*Ra* = 10^3^), a weak global eddy appeared inside the chamber, with upstream flows near the heated left surface and downstream near the cold right surface. The temperature field illustrates the heating of the cavity from the bottom left corner, where the maximum temperature is fixed. Heating occurs like a heat conduction mechanism due to the low value of the Rayleigh number. A raise of *Ra* (=10^4^) results in a strengthening of convective motion and thermal energy transport. As a result, a weak deformation of the convective cell core can be seen with the appearance of the thermal plume along the left vertical surface. Further rise of *Ra* (=10^5^) characterizes a reduction in the heat plume thickness with more significant warming of the chamber, while the major convective cell is degraded with a formation of small recirculation within the upper left corner.

For the uniform heating (see Figure 6), when the temperature on the left and bottom walls is high and constant, one can also reveal an appearance of global clockwise motion and heating of the chamber from these walls. For *Ra* = 10^3^, the major thermal energy transport mechanism is heat conduction, and as a result, the isotherms do not reflect the appearance of a heat plume. The growth of the temperature difference with a rise of *Ra* (=10^4^) illustrates significant liquid motion, and isotherms show the development of ascending and descending thermal plumes along vertical walls. For *Ra* = 10^5^, a global circulation has an elongated convective cell core, and isotherms within the cavity reflect the appearance of the thermal stratification core.

**Figure 7:**
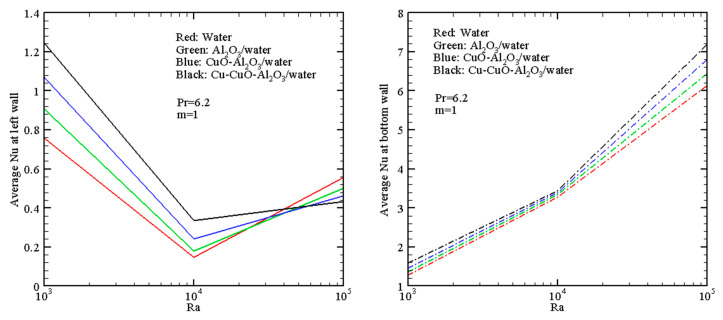
Average Nusselt numbers with *Ra* for different fluids when *m* = 1 (linear heating).

An impact of *Ra* and working fluid structure on *Nu_L_* and *Nu_B_* is shown in Figure 7 for non-uniform heating. These distributions characterize the thermal energy transport augmentation with a transition between the host liquid and ternary hybrid nanofluid through alumina/water nanoliquid and copper oxide–alumina/water hybrid nanoliquid. Essential intensification of the convective energy transfer occurs at the bottom surface due to an interaction between the cold descending fluid flow along the right cold border and the heated bottom border. In the case of the left heated border, the mean Nusselt numbers are not as significant as for the bottom heated wall because there is no essential temperature difference here compared to the lower heated border.

**Figure 8:**
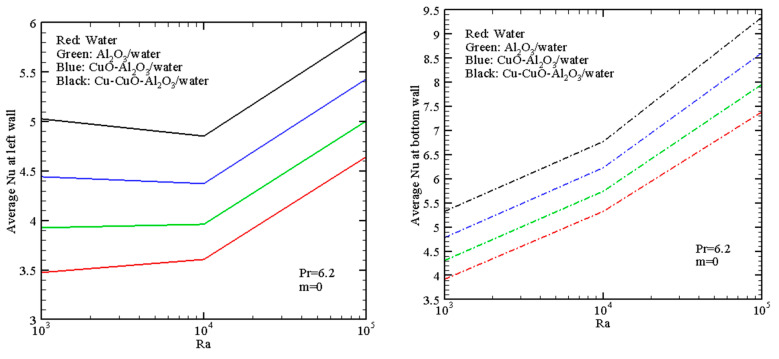
Mean Nusselt numbers with *Ra* for various fluids when *m* = 0 (uniform heating).

For uniform heating (Figure 8), the integral thermal flux from the hot walls is greater than this heat flux for non-uniform wall heating, and as a result, the average Nusselt numbers for *m* = 0 are greater than for the case of *m* = 1. The behavior of *Nu_B_* with the working fluid is the same as in the case of *m* = 1, but for the left border, some differences are obtained. The reason for these differences is the formation of a cold zero temperature at the top left corner with a small recirculation (see Figure 5c) in the case of non-uniform heating.

More detailed outcomes for the average Nusselt numbers for various hybrid nanofluids are shown in Table 5 and Table 6, where one can find that the maximum thermal energy transport strength can be found for Cu–CuO/water.

**Figure 9:**
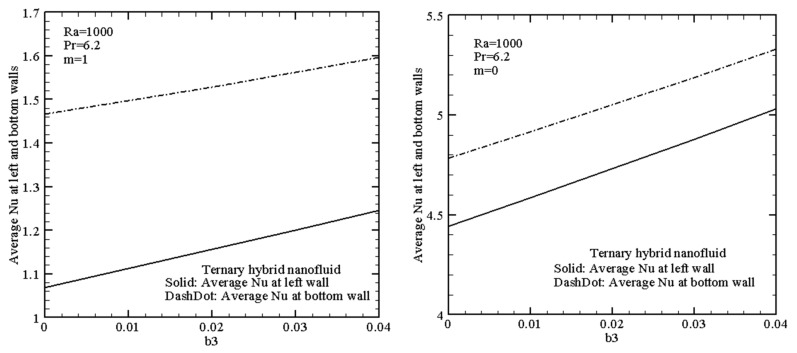
Mean Nusselt number with *b*3 for ternary hybrid nanofluid at *Ra* = 1000 when *m* = 1 (linear heating) and *m* = 0 (constant heating).

Figure 9 demonstrates the impact of the copper nanoadditives concentration on the mean Nusselt number for the non-uniform and uniform heating of ternary hybrid nanofluid at *Ra* = 1000. An increase in the copper nanoadditives concentration reflects a rise in the thermal energy transfer strength of the ternary hybrid nanoliquid, regardless of the considered cases.

## 5. Conclusions

Mathematical modeling of thermal convection of mono-, hybrid-, and ternary nanofluids in a differentially heated chamber with uniform and non-uniform heating from the left vertical and lower horizontal walls has been performed for a wide range of the Rayleigh number and nanoparticle volume fraction. It has been shown that non-uniform heating for high values of the Rayleigh number reflects a formation of secondary recirculation in the upper left corner where the cold temperature is kept. A rise in the Rayleigh number and nanoparticle concentration illustrates the thermal energy transfer enhancement for the ternary nanoliquid, while for the considered hybrid nanofluids, the more effective is Cu–CuO/water. Thus, a growth of the Rayleigh number between 10^3^ and 10^5^ allows increasing the average Nusselt number at the bottom wall by about 76% for the ternary nanoliquid in the case of uniform heating, while in the case of non-uniform heating, one can find a growth of about 4.5 times. A rise in the nanoparticle volume fraction for the ternary nanoliquid for b3 ranges between 0.0 and 0.04, leading to a growth of the average Nusselt number at the bottom wall at about 11% in the case of uniform heating and at about 10% in the case of non-uniform heating. The performed analysis shows that ternary nanofluids can be effective “smart” working fluids that allow intensification of the convective energy transference within the closed chambers.

## Figures and Tables

**Table 1:** Physical attributes of Cu–CuO–Al_2_O_3_/H_2_O.

Property	Ternary Nanosuspension (Cu–CuO–Al_2_O_3_/H_2_O)
Density (ρ)	$\rho_{thnf}=\left(1-b3\right)\left\{\left[\left(1-b2\right)\left\{\left(1-b1\right)\rho_{f}+b1\rho_{s1}\right\}\right]+b2\rho_{s2}\right\}+b3\rho_{s3}$
Dynamic viscosity (μ)	$\mu_{thnf}=\frac{\mu_{f}}{{\left(1-b1\right)}^{2.5}{\left(1-b2\right)}^{2.5}{\left(1-b3\right)}^{2.5}}$
Heat capacity (ρ*c_p_*)	$\begin{array}{l} {\left(\rho c_{p}\right)}_{thnf}=\left(1-b3\right)\times \\ \times \left\{\left[\left(1-b2\right)\left\{\left(1-b1\right){\left(\rho c_{p}\right)}_{f}+b1{\left(\rho c_{p}\right)}_{s1}\right\}\right]+b2{\left(\rho c_{p}\right)}_{s2}\right\}+b3{\left(\rho c_{p}\right)}_{s3} \end{array}$
Thermal expansion coefficient (ρβ)	$\begin{array}{l} {\left(\rho \beta \right)}_{thnf}=\left(1-b3\right)\left\{\left[\left(1-b2\right)\left\{\left(1-b1\right){\left(\rho \beta \right)}_{f}+b1{\left(\rho \beta \right)}_{s1}\right\}\right]+b2{\left(\rho \beta \right)}_{s2}\right\}+\\ +b3{\left(\rho \beta \right)}_{s3} \end{array}$
Thermal conductivity (κ)	$\kappa_{thnf}=\kappa_{hnf}\frac{\kappa_{s3}+2\kappa_{hnf}-2b3\left(\kappa_{hnf}-\kappa_{s3}\right)}{\kappa_{s3}+2\kappa_{hnf}+b3\left(\kappa_{hnf}-\kappa_{s3}\right)}$, where $\kappa_{hnf}=\kappa_{nf}\frac{\kappa_{s2}+2\kappa_{nf}-2b2\left(\kappa_{nf}-\kappa_{s2}\right)}{\kappa_{s2}+2\kappa_{nf}+b2\left(\kappa_{nf}-\kappa_{s2}\right)}$ and $\kappa_{nf}=\kappa_{f}\frac{\kappa_{s1}+2\kappa_{f}-2b1\left(\kappa_{f}-\kappa_{s1}\right)}{\kappa_{s1}+2\kappa_{f}+b1\left(\kappa_{f}-\kappa_{s1}\right)}$

**Table 2:** Physical characteristics of host liquids and nanoadditives [35].

Properties	H_2_O (*f*)	CuO (*s*1)	Cu (*s*2)	Al_2_O_3_ (*s*3)
ρ (kg/m^3^)	997.1	6320	8933	3970
*c_p_* (J/kgK)	4179	531.8	385	765
β (1/K)	21 × 10^–5^	1.8 × 10^–5^	1.67 × 10^–5^	0.85 × 10^–5^
κ (W/mK)	0.613	76.5	401	40

**Table 3:** (**a**). Mesh refinement check with *Nu_L_* and *Nu_B_* for *Ra* = 10^3^, *m* = 1, *Pr* = 6.2, *b*1 = *b*2 = *b*3 = 0.04. (**b**). Mesh refinement check with *Nu_L_* and *Nu_B_* for *Ra* = 10^3^, *m* = 0, *Pr* = 6.2, *b*1 = *b*2 = *b*3 = 0.04.

(**a**)				
	**Elements Number**	**Nodes Number**	***Nu_L_***	***Nu_B_***
Extremely Coarse	192	145	1.2465	1.5958
Extra Coarse	354	250	1.24678	1.59534
Coarser	540	361	1.24632	1.59534
Coarse	1012	645	1.24626	1.5953
Normal	1510	930	1.24636	1.59518
Fine	2516	1475	1.24628	1.59514
Finer	6536	3731	1.24594	1.59548
Extra fine	16,946	9374	1.2458	1.59564
Extremely fine	26,352	14,077	1.24578	1.59566
(**b**)				
	**Elements Number**	**Nodes Number**	***Nu_L_***	***Nu_B_***
Extremely Coarse	192	145	2.3938	2.707
Extra Coarse	354	250	2.7683	3.0627
Coarser	540	361	2.9665	3.2585
Coarse	1012	645	3.3523	3.6427
Normal	1510	930	3.5601	3.8578
Fine	2516	1475	3.7483	4.048
Finer	6536	3731	4.4309	4.731
Extra fine	16,946	9374	5.0301	5.3296
Extremely fine	26,352	14,077	5.0311	5.3294

**Table 4:** Statistics of effective mesh type (extra fine).

Grid Information	Grid Statistics
Number of elements	
Quads	1200
Edge elements	600
Triangles	15,746
Average element quality	0.8028
Number of Nodes	9374
Vertex elements	4

**Table 5:** Mean Nusselt numbers with *Ra* for different nanosuspension when *m* = 1 (linear heating).

	CuO–Al_2_O_3_/Water		Cu–Al_2_O_3_/Water		Cu–CuO/Water	
*Ra*	*Nu_L_*	*Nu_B_*	*Nu_L_*	*Nu_B_*	*Nu_L_*	*Nu_B_*
10^3^	1.0689	1.46628	1.07054	1.47114	1.07014	1.47836
10^4^	0.24	3.3962	0.23986	3.4116	0.23772	3.4418
10^5^	0.46208	6.8034	0.4619	6.818	0.46546	6.8516

**Table 6:** Mean Nusselt number with *Ra* for various hybrid nanofluids at *m* = 0 (uniform heating).

	CuO–Al_2_O_3_/Water		Cu–Al_2_O_3_/Water		Cu–CuO/Water	
*Ra*	*Nu_L_*	*Nu_B_*	*Nu_L_*	*Nu_B_*	*Nu_L_*	*Nu_B_*
10^3^	4.4442	4.7838	4.4544	4.7968	4.463	4.8119
10^4^	4.371	6.2361	4.3833	6.2559	4.3984	6.2847
10^5^	5.4299	8.612	5.4429	8.6425	5.4635	8.6863

## Data Availability

Not applicable.
